# Can Persistence Hunting Signal Male Quality? A Test Considering Digit Ratio in Endurance Athletes

**DOI:** 10.1371/journal.pone.0121560

**Published:** 2015-04-08

**Authors:** Daniel Longman, Jonathan C. K. Wells, Jay T. Stock

**Affiliations:** 1 Department of Archaeology and Anthropology, University of Cambridge, Cambridge, United Kingdom; 2 Childhood Nutrition Research Centre, UCL Institute of Child Health, London, United Kingdom; University of Goettingen, GERMANY

## Abstract

Various theories have been posed to explain the fitness payoffs of hunting success among hunter-gatherers. ‘Having’ theories refer to the acquisition of resources, and include the direct provisioning hypothesis. In contrast, ‘getting’ theories concern the signalling of male resourcefulness and other desirable traits, such as athleticism and intelligence, via hunting prowess. We investigated the association between androgenisation and endurance running ability as a potential signalling mechanism, whereby running prowess, vital for persistence hunting, might be used as a reliable signal of male reproductive fitness by females. Digit ratio (2D:4D) was used as a proxy for prenatal androgenisation in 439 males and 103 females, while a half marathon race (21km), representing a distance/duration comparable with that of persistence hunting, was used to assess running ability. Digit ratio was significantly and positively correlated with half-marathon time in males (right hand: *r* = 0.45, *p*<0.001; left hand: *r* = 0.42, *p*<0.001) and females (right hand: *r* = 0.26, *p*<0.01; left hand: *r* = 0.23, *p* = 0.02). Sex-interaction analysis showed that this correlation was significantly stronger in males than females, suggesting that androgenisation may have experienced stronger selective pressure from endurance running in males. As digit ratio has previously been shown to predict reproductive success, our results are consistent with the hypothesis that endurance running ability may signal reproductive potential in males, through its association with prenatal androgen exposure. However, further work is required to establish whether and how females respond to this signalling for fitness.

## Introduction

### Hunting and reproductive success

The high value placed by females on male ability to acquire resources has been well documented [1]. This is evident in pre-industrial human societies, where males exhibit a positive relationship between status and number of surviving offspring. Such observations have been made across the continents of Africa, South America and Asia [2–4]. It has been suggested that the same is true of contemporary Western society; increasing income has been shown to have a significant effect on male reproductive success and desirability as a marriage partner [1,5–7].

Prior to agriculture, hunting may have represented an important means by which male resourcefulness could be demonstrated. Food is inexorably linked to status in many cultures around the world [8], and there is evidence that superior hunters enjoy social prestige within the community [9]. Indeed, successful hunters have been shown to enjoy higher reproductive success [10,11]. Hunting may therefore be motivated by male-male competition [12]. Smith [4] has reviewed quantitative links between hunting ability and fitness-related factors such as fertility, offspring survivorship and number of mates, and qualitative links between hunting success and knowledge of hunting ability within communities. However, it is not clear whether it is the high social status of hunters (which reflects their ability to acquire resources), or the actual resources they obtain, that provides the mechanism linking hunting success with reproductive fitness.

Theories posed to explain the fitness payoffs of hunting success include the "direct provisioning" hypothesis, which asserts that successful hunters are more able to share food with their mate and children, thereby enhancing fertility and offspring survivorship through physiological mechanisms. In accordance with this theory, the offspring of successful hunters may indeed be better nourished [13]. Recent endocrine data from subsistence populations has revealed that while testosterone levels increase upon a successful kill, this increase is not associated with the number and size of kills or the presence of an audience beyond the hunter's family [14]. These findings are consistent with the direct provisioning model; meat provisioning enhances reproductive success either directly [11], or indirectly through political alliances and other benefits stemming from the community's desire to retain a successful hunter as a neighbour [15–18]. Although this theory has intrinsic appeal, the egalitarian organisation of many forager societies ensures that this nepotistic distribution of food is not consistently observed [3]. While some male hunters are more effective than others, meat is widely distributed throughout the group. As a result most of the food consumed is caught by a man outside one's own nuclear family, such that the hunter's own family may receive no more meat than anyone else [3]. Such sharing has been attributed to a means of maintaining social identity, and has been considered a defining characteristic of hunter-gatherer behaviour [19]. Food sharing also serves as a mechanism to deal with fluctuations in food availability [20]. A hunter may exchange a short-term food surplus for receipt of food in the future should his own hunting efforts fail; a phenomenon known as the reciprocal altruism [21]. When food resources are scarce and hunting success unpredictable, food sharing is prevalent as a type of culinary insurance policy, as seen in the Inuit of Akalivik [22]. However, food donations may not always be reciprocated [23,24]. Smith [4] is doubtful as to the efficacy of this proposal due to the lack of a system of conditional reciprocity linked to meat provisioning. Thus, perhaps counter-intuitively, there is very little clear evidence that the meat produced by hunting is the primary mechanism underpinning the reproductive fitness of hunters. The 'having' theories do not provide adequate explanation.

A third hypothesis, based on Zahavi's "handicap principle" [25], is that hunting success acts as a reliable signal of enviable underlying traits such as athleticism, intelligence or altruism. Successful hunters benefit the community through the provisioning of public goods, an act which enhances their reputation for generosity. The pursuant social standing thereby attained may be attractive to women due to benefits of association such as protection [26]. As such, hunting returns may be exchanged for the future fitness-enhancing benefits of increased social capital or status [27–29]. This theory differs from those previously mentioned in that it need not necessarily be the resource acquisition itself which promotes the reproductive fitness ('having'), but the signal such resourcefulness conveys of underlying male quality ('getting'). The distribution of acquired meat beyond one's own family unit would also ensure that any such signal was more widely observed [30]. Indeed, hunters are aware that others will know who acquired the meat [31,32]. Male hunting may not then be motivated purely by consequent meat consumption [12,33]. Such signalling would be reliable if it were costly to the signaller, such that an individual lacking in a certain trait would be unable to afford to mimic the signal.

The hypothesis that hunting may act as a signal has been addressed by several authors [30,34,35], but it is as yet unclear what the underlying mechanism may be. In other words, what makes the signal a reliable index of fitness. We first review the physical traits that contribute to hunting success, and then consider potential mechanisms whereby these traits might signal reproductive potential.

### Hunting success and physical skills

The hunting of mid-sized animals using hunting technologies such as spears favours a method of disadvantaging or weakening the animal before approaching for the kill. This is because the killing range of spears is very limited, and being close enough to kill a mid-sized animal would be to risk injury to the hunter. Persistence hunting offers such a method, and may be a very effective means of food acquisition under certain conditions. Persistence hunting is a technique by which hunters track and chase prey to the point of prey exhaustion or hyperthermia, often during the hottest part of the day. This technique has been observed in the Kalahari in Africa, and the Tarahumara tribe of Northern Mexico, who have been reported chasing deer until they collapse before strangling them by hand [36,37]. Other animals targeted in this way include steenbok, gemsbok, duiker, caracal, cheetah, kudu and eland [38,39]. During the hunt, chases of up to 35km have been documented. Persistence hunting is useful from the point of view of the early hunter for three reasons; it is relatively low risk, easy for a fit human with animal tracking skills, and requires low metabolic cost relative to potential pay-off [40].

Endurance running may therefore represent a valuable component of hunting success. The evolution of human endurance running ability has attracted attention previously [41,42]. This followed observations that, in comparison with other cursorial animals, humans perform very well over long distances. We are unique amongst primates in possessing the ability to run distances of several kilometres using aerobic metabolism [41], with many amateur human runners able to sustain speeds of 5m/s [40]. This is fast compared with specialised quadrupedal cursors; a dog with a similar mass to a human (65kg) has a trot-gallop transition speed of 3.8m/s, and can then only sustain a gallop for a maximum of 15 minutes under ideal conditions [43]. The same is true of horses, which is surprising as they have been selectively bred for running ability [44]. Human runners can easily cover distances exceeding 10km a day; comparable with hunting dogs and hyenas which run to scavenge and hunt [40]. Consequently, the physical capacity for endurance running appears have been selected for in our genus. Hunting may well have provided the primary selective pressure; evidence for hominin carnivory dates back to approximately 2.5Ma [45]. The ability to run long distances to either hunt or scavenge may have improved the chances of acquiring meat [40,46]. But as described above, if meat was widely shared, how might hunting ability translate into fitness payoffs?

Endurance running might benefit male fitness if it acted as a reliable signal of reproductive potential. Since testosterone is widely associated with reproductive success [47,48], an association between testosterone and endurance running would make running prowess a reliable signal of male reproductive potential. Recent work has reported associations between sporting ability and a marker for foetal testosterone exposure, the 2D:4D ratio [49,50]. This developmental association between testosterone and physical abilities makes it a viable candidate as a signalling mechanism.

### The ontogenetic development of digit ratio variability, and significance for reproductive success

The sexually dimorphic digit ratio (2D:4D), first noted by Baker [51], is linked to prenatal androgenisation [52]. The presence of significant sexual dimorphism in the digit ratio of deceased human foetuses suggests 2D:4D ratio is established early in prenatal development [53]. Analysis of amniotic fluid provided direct evidence for a relationship between foetal hormones and digit ratio [54]. This followed indirect reports stemming from homeobox genes [55,56] and congenital adrenal hyperplasia [57,58]. Although the relationship between high digit ratio and prenatal testosterone exposure has been questioned [59], and may be confounded by sexual dimorphism in body size [60], the majority of evidence suggests that digit ratio is a suitable instrument for investigating effects of prenatal androgen exposure on subsequent phenotype [52]. As a result digit ratio is increasingly used as a proxy for foetal hormone environment—to investigate early life predictors of later phenotype, and sex differences therein.

Digit ratio predicts reproductive success in both men and women once the effects of age and population have been removed [61]. Mechanistically, low digit ratios are associated with higher sperm counts and testosterone levels in men, and, conversely, high digit ratios are linked with high oestrogen and luteinising hormone in women [62]. Furthermore, males exhibit a negative relationship between digit ratio and other correlates of reproductive success such as preferred number of children, strength of sex-drive, ease of achieving sexual excitement [47] and sperm numbers per ejaculate [62]. Thus, digit ratio variability emerging early in life has significant implications for fitness.

Digit ratio is negatively correlated with physical prowess across a range of sporting disciplines from slalom skiing [63] to football [64]. Digit ratio has also been tentatively correlated to middle-distance running ability, albeit with a small sample size at distances of less than 4 miles [65]. This study by Manning et al was the first to discuss the relationship between digit ratio and running within the context of persistence hunting. Digit ratio was found to explain a larger portion of the variance in endurance running than expected (up to 25%); explaining more variation than other sports [66] and running over shorter distances [67,68]. As such, the literature supports the view that while 2D:4D does predict running speed, the predictive power increases from sprinting events (1–2%) to events of up to 4 miles in length (20–25%).

We therefore sought to test the hypothesis that physical prowess at endurance running is associated with this putative marker of testosterone exposure. This was performed with a larger sample and distance than previously reported. A female sample was included, as the relationship between female digit ratio and running ability is as yet unknown. Since men undertake the majority of hunting [69], we further predicted that the association between digit ratio and endurance running prowess would be stronger in males than females. Such a sex-difference would suggest that the selective pressure of endurance running has shaped running prowess to be a stronger signal of reproductive potential in males than females. Through such preferences, males would then convert their physical prowess into fitness gains. Our study therefore tests a potential mechanism whereby ‘getting’ meat could translate into male fitness returns.

## Materials and Methods

Athletes (*N* = 542; m = 439, f = 103) were recruited at the 2013 Robin Hood Half Marathon (21km), held on September 29^th^. Athletes received an email explaining the study prior to race day, and were invited to take part following the race. Weather conditions were clear skies, with a mean day-time temperature 14°C. The race is a high-profile event, with course records of 61:38 and 73:32 for men and women respectively. Participants ranged between the ages of 19 and 35, and were all Caucasian. The half-marathon distance was chosen due to its appropriateness to evolutionary hunting-associated running [36]. Race time over 21km was the performance indicator. While this is a measure of speed, the half-marathon distance is considered to be very different from pure speed events such as the 100m sprint in terms of the energy systems utilised. While the 100m sprint utilises the anaerobic system to supply ATP at high rates, prolonged endurance races such as the half-marathon are dependent upon the more sustainable aerobic energy system and lipid metabolism [70–74]. Half-marathons are often used to investigate the effects of endurance exercise on physiology [75–78], so we are confident the event may be used to consider endurance running ability.

All participants participating in the same race, ensuring standardisation of not only distance, but also of time-affecting factors such as weather conditions, time of day and race-course elevation profile. All competitors wore small electronic chips which uniquely identified them as they crossed electronic mats at the start and finish lines, guaranteeing accurate race timings.

Photocopies were taken of athletes' hands upon finishing the race, and measurements of digit ratio were made at a later date. It is appreciated that ratios obtained from photocopies and from direct measurements should not be combined within one study [79], so all measurements were taken from photocopies, with the same machine being used for all copies. This method was chosen due to ease of use and speed in facilitating a large sample size. Digit ratio was measured using Mitutoyo electronic callipers, reading to 0.01mm. Measurements were taken twice from each photocopy to check repeatability. Digit ratios from the first measurement were strongly correlated with the lengths recorded from the second measurement for all individual participants (all r > 0.95). In addition, the means of individual right hand and left hand digit ratios were significantly correlated (male r = 0.88, P < 0.001; female r = 0.762, P < 0.001). The precision of each measurement was found using the method of Bland & Altman [80]; male right 2D = 2.52; male right 4D = 2.21; male left 2D = 2.36; male left 4D = 2.07; female right 2D = 2.24; female right 4D = 2.04; female left 2D = 1.93; female left 4D = 2.42, all measurements to 2 decimal places. These associations gave confidence in the reliability of the hand-specific measurements of digit ratio, and therefore in the conclusions subsequently drawn.

Ethical approval for the project was granted by the University of Cambridge Human Biology Ethics Committee.

## Statistics

Crude associations between digit ratio and race time were explored in each sex using correlation analysis. The difference between the male and female correlation coefficients was analysed using the Fisher r-to-z transformation and subsequent comparison of z-scores [81,82]. Multiple linear regression was used to analyse the sex difference in the relationship between digit ratio and race time. Sex was coded as 0 (female) versus 1 (male), and an ‘Interaction’ term was constructed by multiplying sex code by digit ratio. Each of sex, ‘interaction’ and digit ratio were then used as predictors of race time.

## Results

### Descriptive statistics

A description of the male and females samples is given in Table 1. Males were older, and had significantly lower right and left digit ratios than females.

**Table 1:** Descriptive characteristics of the samples.

	Males (*n* = 439)		Females (*n* = 103)	
	Mean	SD	Mean	SD
Age (y)	31.7	4.93	28.8	4.58
2D:4D ratio right	0.97	0.033	0.98	0.028
2D:4D ratio left	0.94	0.036	1.01	0.035
Race time (s)	6946	1313	7002	926

**Fig 1:**
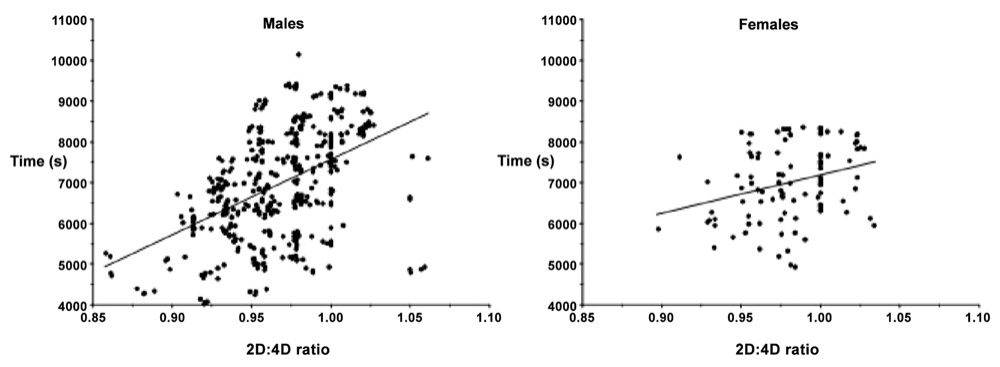
Scatter plot of male and female right hand 2D:4D ratio versus half marathon performance (s). The steeper male gradient is visible.

Among the male subsample there was a significant positive correlation between right and left hand 2D:4D ratio and half-marathon time (right *r* = 0.45, *p* < 0.001; left *r* = 0.42, *p* < 0.001). When age was controlled for the correlation strengthened (right *r* = 0.47, *p* < 0.001; left *r* = 0.43, *p* < 0.001). The same was true among the female subsample (right hand *r* = 0.26, *p* < 0.01; left hand *r* = 0.23, *p* = 0.02), with the correlation strengthening when age was controlled for (right *r* = 0.29, *p* < 0.01; left *r* = 0.26, *p* < 0.01). These positive correlations can be seen below in Fig. 1. Note the steeper regression line in the male subsample compared to the female subsample.

Regression analysis revealed that men performed better than women, higher levels of performance were associated with lower digit ratio, and digit ratio increases performance more significantly in men than women (*p*<0.05). Age was not a significant contributor in the regression model, so was removed. See Table 2 for a summary of the results.

**Table 2:** Regression of half-marathon time in seconds on digit ratio (right in model 1, left in model 2), sex and sex-ratio interaction (right in model 1, left in model 2).

Step	Predictor	β	*t*	*p*
	**Model 1**			
1	Age	-.057	-1.317	.188
Δ *R*2 = .003, *F* Change (1,540) = 1.736, *p* = .188				
2	Age	-.007	-.186	.853
	Sex Code	-2.692	-2.058	.040
	Right Interaction	2.725	2.104	.036
	Right ratio	.249	2.410	.016
Δ *R*2 = .203, *F* Change (3,537) = 45.692, *p* < .001				
**Model summary:** *F*(4,537) = 34.810, *p* < 0.001, *R*2 = .206				
	**Model 2**			
1	Age	-.057	-1.317	.188
Δ *R*2 = .003, *F* Change (1,540) = 1.736, *p* = .188				
2	Age	-.030	-.734	.463
	Sex Code	-2.419	-2.174	.030
	Left Interaction	2.561	2.427	.016
	Left ratio	.248	2.201	.028
Δ *R*2 = .172, *F* Change (3,537) = 37.239, *p* < .001				
**Model summary:** *F*(4,537) = 28.451, *p* < 0.001, *R*2 = .175				

## Discussion

It was hypothesised that 2D:4D ratio correlates with endurance running performance over the half-marathon distance. This hypothesis has been supported. The male effect sizes are similar to those of Manning et al. [65], which is consistent with the theory that digit ratio explains more variation in endurance running than it does in shorter running events or other sports. The link between digit ratio and maximal oxygen uptake may well relate to these observations [83].

**Fig 2:**
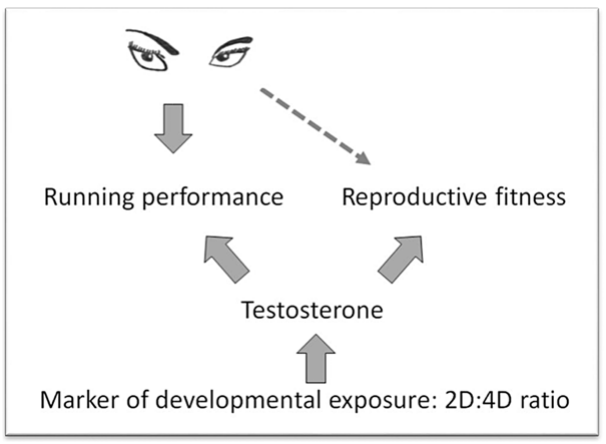
Conceptual diagram outlining a potential mechanism by which hunting success and running performance act as a signal of reproductive fitness.

A marker of testosterone exposure is therefore associated with running ability, which ethnographic evidence has shown to be an important attribute for hunting [37]. As testosterone (including the 2D:4D ratio) has been consistently associated with reproductive success [47], this relationship between endurance running ability and digit ratio provides mechanistic evidence in support of the hypothesis that running prowess could act as a reliable signal for male reproductive potential. A conceptual diagram outlining this potential mechanism by which hunting success and running performance could act as a signal of reproductive fitness is given below in Fig. 2.

As a positive relationship between hunting ability and male reproductive success has been reported [2,3], it may be that women are attracted to men with the capacity to ‘get’, rather than those who ‘have’. This may be a consequence of the egalitarian organisation of many forager societies, which ensures that meat is widely distributed throughout the group [3]. It is acknowledged, however, that this study has not demonstrated that women are in fact differentially attracted to faster male runners, or that this is the only mechanism potentially linking hunting success with reproductive success. Of course, hunting ability and reproductive success may both be correlated with another independent variable which is itself a cause of higher reproductive success, independent of the male's hunting abilities [4].

Although both sexes exhibited a statistically significant positive correlation between right and left hand 2D:4D ratio and time taken to complete a half-marathon running race, the relationship exhibited by males was significantly stronger than that of females. As such, the secondary hypothesis was also supported, suggesting that the ability of running ability to signal reproductive potential has been under stronger selection in males than in females.

Testosterone is not only a link between digit ratio and endurance running ability, but also an important mediator of the male reproductive effort. Behaviourally, testosterone plays an important role in producing sex drive [84] and mediating confidence and assertiveness in social situations [85] – qualities deemed beneficial in the male mating effort. Physiologically, testosterone serves to promote muscle growth [86,87], providing an advantage in male-male combat situations [48,88]. Muscularity is also a sexually attractive trait; more muscular men to report a greater number of sexual partners and younger age at first conception [89,90] and more offspring sired [91].

Muscularity per se, however, could be disadvantageous as a signal of male fitness in a hot environment, as the link between muscle mass and metabolic rate means that a larger muscle mass could induce overheating. Additionally, the high metabolic cost of muscle tissue would not be well suited to environments prone to famine. Running ability may therefore represent a signal of reproductive fitness that is better suited to a hot stochastic environment, where it is furthermore compatible with immediate benefits, given that hunting promotes food acquisition.

Comparative analyses of Olympic winning times and world records has lead some authors to conclude that women will outcompete men in the marathon by the end of the 20th century and over 100m in the mid 22nd century [92,93]. However, caution is required as the initial greater rate of improvement in female performances is most likely due to historical considerations such as the later social acceptance and inclusion of female distance running in major events such as the Olympic Games [94]. The gender difference is now believed to have reached a plateau [95], with the remaining disparity explicable by biological differences between males and females [96]. While it has been suggested that the gender difference in performance disappears as distances increase beyond that of the marathon [97,98], several studies have reported that such distances exhibit no change in relative ability [99]. Indeed, performances between the sexes may even diverge [100,101]. Although it is possible that females improve relative to males with distance, such considerations are not deemed to be relevant to this study as ultra-marathon distances are not applicable to the evolutionary pressures applied by persistence hunting.

To conclude, this investigation has shown that both male and female digit ratios are significantly and negatively correlated to ability in endurance running of a distance/duration comparable with that of persistence hunting (a positive correlation between digit ratio and half marathon time). The relationship was stronger for males than females, suggesting that males faced greater evolutionary pressure to develop endurance running capabilities. We suggest that hunting ability might therefore act as a reliable signal of male fitness, in addition to its function of calorie acquisition. Consequently, the ability to 'get' meat may contribute to the positive relationship between hunting ability and male reproductive success. Our work has used sporting ability as a proxy for hunting prowess; further work is now required to test this hypothesis in hunting societies.
